# Morphometric analyses of canine blood microfilariae isolated by the Knott’s test enables *Dirofilaria immitis* and *D. repens* species-specific and *Acanthocheilonema* (syn. *Dipetalonema*) genus-specific diagnosis

**DOI:** 10.1186/1756-3305-6-48

**Published:** 2013-02-25

**Authors:** Johannes Magnis, Susanne Lorentz, Lisa Guardone, Felix Grimm, Marta Magi, Torsten J Naucke, Peter Deplazes

**Affiliations:** 1 Kleintierklinik in Iffezheim, An der Rennbahn 16a, 76473, Iffezheim, Germany; 2 Parasitus Ex e.V, Vollbergstrasse 37, 53859, Niederkassel, Germany; 3 Department of Veterinary Science, University of Pisa, viale delle Piagge 2, 56124, Pisa, Italy; 4 Institute of Parasitology, University of Zurich, Winterthurerstr. 266a, CH-8057, Zurich, Switzerland

**Keywords:** Canine blood microfilariae, Laboratory diagnosis, Size of microfilariae

## Abstract

**Background:**

Considering the increasing importance of small animals travel medicine and the spread of filariae with zoonotic potential to non-endemic European areas, routine filarial diagnosis in dogs is becoming important. *Dirofilaria immitis*, *D. repens*, *Acanthocheilonema dracunculoides* and *A. reconditum* are the most common canine filarial nematodes presenting blood circulating microfilariae (mf) which can be differentiated to species level by the acid phosphatase activity patterns or by PCR. Available data on the size of the mf vary considerably in the literature. The aim of this study was to validate morphometric criteria for filarial identification in blood samples of dogs after concentration of mf with the modified Knott’s technique.

**Methods:**

Morphometric analysis of 10 mf from samples identified to species level by acid phosphatase activity and partially confirmed by PCR were performed with specimens from 377 dogs.

**Results:**

The mean length and width of *D. immitis* mf from 60 dogs were 301.77±6.29 μm and 6.30±0.26 μm, of *D. repens* mf from 171 dogs 369.44±10.76 μm 8.87±0.58 μm, of *A. dracunculoides* mf from 133 dogs 259.43±6.69 μm and 5.09±0.47 μm and of *A. reconditum* mf from 13 dogs 264.83±5.47 μm and 4.63±0.52 μm.

For a subset of 30 samples, morphometric analysis was repeated with identical results in two laboratories. Furthermore, the size of mf concentrated and fixed by the Knott’s technique was shown to be stable over 105 days.

**Conclusions:**

The Knott’s test enables to clearly distinguish between *D. immitis*, *D. repens* and *Acanthocheilonema* spp. However, due to the overlapping size ranges of *A. dracunculoides* and *A. reconditum*, biochemical or molecular methods are required to distinguish these two species.

## Background

In Europe the most common canine filarial species presenting blood circulating microfilariae (mf) are *Dirofilaria immitis*, *D. repens*, *Acanthocheilonema* (syn. *Dipetalonema*) *dracunculoides* and *A.* (syn. *Dip.*) *reconditum*[1]. They are transmitted by haematophagous arthropods: *Dirofilaria* spp. by mosquitoes, *A. reconditum* by fleas and lice and *A. dracunculoides* by ticks. *D. immitis*, the aetiological agent of canine heartworm disease, is the most pathogenic species for dogs: adults live in the right side of the heart and in the pulmonary artery, causing pulmonary hypertension and congestive heart failure. Adults of *D. repens* live in the subcutaneous tissue, occasionally causing dermatological problems [2]. *A. reconditum* and *A. dracunculoides* live in the peritoneal cavity and adipose tissue and are less pathogenic [3]. However, *D. immitis* and *D. repens* are considered emerging agents of parasitic zoonoses in Europe with expanding ranges [1], and *A. reconditum* has occasionally been reported as a zoonotic agent [4].

All these species release mf in the blood of their final hosts, and diagnosis of canine filariosis is mainly based on the detection of circulating mf. Specific identification of these stages is essential for an accurate diagnosis and for choosing the appropriate treatment [5,6]. Data on length and width of mf reported in the literature vary considerably. The objective of this study was to validate morphometric criteria for species or genus identification of mf concentrated by the modified Knott’s technique, a classical, inexpensive and widely used method.

## Methods

Canine EDTA blood samples containing mf (n=379) were collected between 2006 and 2011 by different parasitological laboratories in Europe and sent to Parasitus Ex e.V. in Niederkassel (lab 1). The samples were taken from dogs from Spain (73), Portugal (102), Greece (6), Italy (30), Romania (3), Hungary (141), Bulgaria (1), Turkey (2) and France (1); for 19 samples the dog origin was unknown. Dogs of both sexes and various age groups were sampled; exact age determination was impossible for most dogs due to their unknown history. The modified Knott’s technique was applied to concentrate and to detect mf. One ml of EDTA blood was mixed with 9 ml of 2% formalin in a 15 ml tube and centrifuged for 5 minutes at 500 × g. The supernatant was poured off, and 2 × 10 μl of the sediment was transferred to a slide and covered with a coverslip.

Morphometric analyses of the mf were conducted with standard diagnostic microscopes equipped with calibrated measuring eyepieces at a final magnification of 200–400 x. Body length and diameter, and the form of the front end and the tail of ten randomly selected mf were determined. To demonstrate the reliability of the measurements, a subset of 30 samples was repeatedly tested at the Institute of Parasitology, University of Zürich, Switzerland (lab. 2, *D. immitis, D. repens* and *A. dracunculoides*) and at the Department of Veterinary Science, University of Pisa, Italy (lab. 3, *A. reconditum*).

To estimate size variations in relation to time, microfilariae in two samples concentrated by the modified Knott’s technique were measured repeatedly after intervals of 30 minutes, 2 hours, 6 hours, 24 hours, 7 days, 18 days, 31 days, 45 days, and 105 days (storage of concentrated material at 4°C) by two independent operators in labs 1 and 2.

All samples had been identified to species level by demonstrating the characteristic acid phosphatase activity patterns [5]. The Leucognost SP® kit was used according to the manufacturer’s recommendations to demonstrate acid phophatase activity patterns in the mf [6]. The fourteen *A. reconditum* positive samples were additionally stained histochemically [5], as no experience with Leucognost SP® exists for this species as yet.

Heartworm antigen detection tests were carried out on all blood samples (FASTest® Hw Ag.; MegaCor or DiroCHEK®, Synbiotics). To confirm species identification by acid phosphatase activity, 6 samples with *D. immitis*, 4 with *D. repens*, 7 with *A. dracunculoides* and 14 with *A. reconditum* were investigated by PCR [7], and sequencing confirmed the species identification in all cases.

## Results and discussion

**Figure 1:**
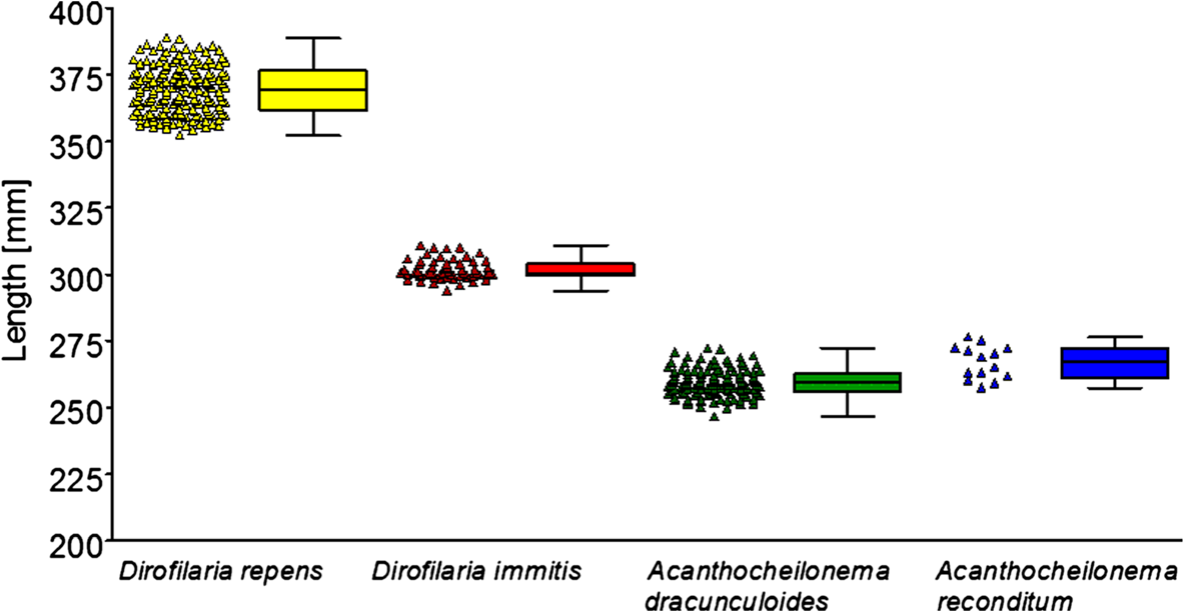
**Mean length (μm) of microfilariae determined after fixation with the modified Knott’s technique. **Measurements were conducted for 377 dogs with confirmed filarial infections (histochemical staining and PCR in part of the cases); each indicator corresponds to a dog sample, for which 10 microfilariae were measured.

The morphometric results (including standard deviations) are shown in Table 1 and in Figure 1. The mean length of *D. immitis* mf was 302 μm, the mean width was 6 μm, with a conical front end and a straight rear end.

**Table 1:** Measures (μm) of microfilariae determined after fixation with the modified Knott’s technique

**Microfilariae species identified by histochemical staining and partially confirmed by PCR**^**a**^	**Total number and origin of dogs**	**Mean length ± standard deviation (μm)**	**Mean width ± standard deviation (μm)**
*Dirofilaria immitis*	60 (GR 1; IT 6; PT 33; ES 16; TR 2; UO 2)	301.77 ± 6.29	6.30 ± 0.26
*Dirofilaria repens*	171 (BG 1; FR 1; GR 5; IT 10; RO 3; ES 2; HU 141; UO 8)	369.44 ± 10.76	8.87 ± 0.58
*Acanthocheilonema dracunculoides*	133 (PT 69; ES 55; UO 9)	259.43 ± 6.69	5.09 ± 0.47
*Acanthocheilonema reconditum*	13 (IT 13)	264.83 ± 5.47	4.63 ± 0.52

Mean length and mean width (μm) of microfilariae determined after fixation with the modified Knott’s technique of confirmed single filarial infections in 377 dogs.

IT, Italy; ES, Spain; GR, Greece; PT, Portugal; RO, Romania; HU, Hungary; BG, Bulgaria; TR, Turkey; FR, France; Unknown Origin: UO.

^a^ PCR confirmation for 6 samples positive for *D. immitis*, 4 for *D. repens*, 7 for *A. dracunculoides* and 6 for *A. reconditum* infections.

Microfilariae of *D. repens* were 369 μm in length and 9 μm in width, with a conical front end and curved caudal end. For *A. dracunculoides,* the mf length was 259 μm and the width 5 μm. The front was round, the end of the tail was straight. Mf of *A. reconditum* had a mean length of 265 μm and a mean width of 5 μm. The front end was blunt, the rear end showed a small hook. In one blood sample, a mixed infection with *D. immitis* and *A. dracunculoides* was detected. In this case, circulating *D. immitis*-antigens could be demonstrated and, after staining with the Leucognost SP® kit, most of the mf were identified as *A. dracunculoides* and a lower number as *D. immitis*. However, only *A. dracunculoides*-DNA was detected by PCR in this sample. Another dog was positive for *D. repens* and *A. reconditum*; in this case histochemical staining and PCR identified both species. These two samples were excluded from the morphometric analyses (Table 1).

**Figure 2:**
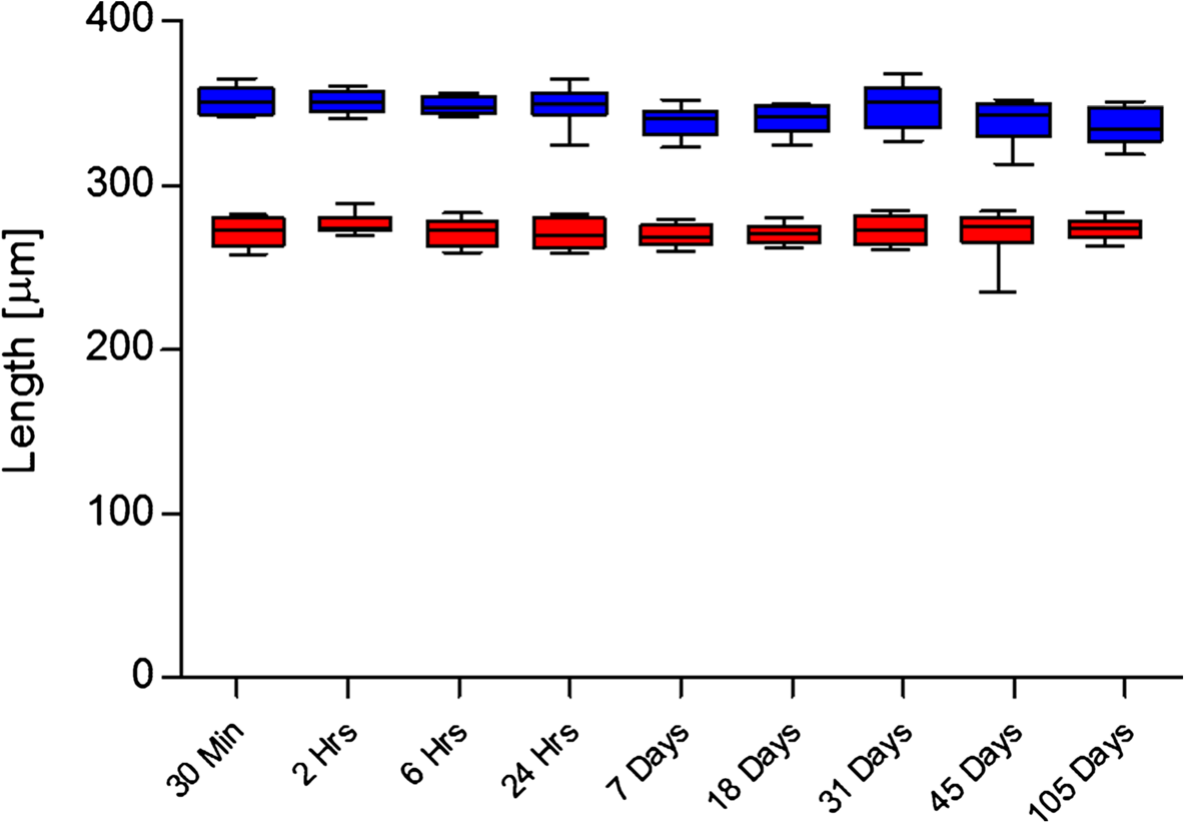
**Mean length of *Dirofilaria repens *and *Acanthocheilonema dracunculoides *microfilariae over time. **Length of microfilariae (n=10) of *Dirofilaria repens* (blue, above) and *Acanthocheilonema dracunculoides* (red, below) after formalin fixation with the modified Knott’s technique over a period of 105 days.

No significant differences of the morphometric data were observed for the mf in the 30 samples retested in labs 2 and 3 (data not shown). Furthermore, no significant size differences were observed over time (30 minutes to 105 days) in formalin fixed samples (Figure 2): the stability of fixed microfilariae is of practical importance as it allows transport of samples to a specialized laboratory if needed.

In accordance with the expected acidic phosphatase activity patterns [5,8,9], *D. immitis* mf displayed two spots (excretory and anal pores), *D. repens* one spot (anal pore) and *A. dracunculoides* three areas of enzymatic activity (anal pore, internal body, excretory pore) when stained with the Leucognost SP® kit. Using this kit, acid phosphatase activity could not be demonstrated in any of the *A. reconditum* mf. A technical failure of the kit could be excluded since *D. immitis* specimens were included in the staining runs and always gave results as expected. When stained histochemically [5], *A. reconditum* mf showed a diffuse light red pattern. The reason why *A. reconditum* mf were not stained by Leucognost SP® is not clear, and more experience with this kit applied to *A. reconditum* is needed. Circulating *D. immitis* antigens could be demonstrated in all samples containing mf of this species. All other samples, except for one case where *A. dracunculoides* mf were detected, were negative with this test. PCR [7] and sequencing confirmed the species identification in all cases.

The measures found in our study agree well with the results of comparable studies [5,10,11]. In a survey of 351 dogs, mf of *D. immitis* were reported to be 311.3 ± 9.5 μm long and 5.96 ± 0.15 μm wide, *D. repens* 366.2 ± 12.1 × 6.40 ± 0.3 and *A. reconditum* 265.2 ± 10.1 × 5.01 ± 0.49 [10]. The latter species has recently been reported with a mean length of 273.1±9.4 and a mean width of 5.4 ± 0.2 [11]. In another study, the length of *D. repens* mf isolated by the modified Knott’s technique from blood samples of 8 Romanian dogs had been reported to vary between 300.3 ± 38.0 and 342.8 ± 18.1 [12]. The obvious difference to our measurements cannot be fully explained. However, the authors solely used morphological criteria for species identification without confirmation by additional histochemical or molecular methods. Regarding *A. dracunculoides,* our results correspond to a study [9] in which the mean size of 150 microfilariae from 14 positive dogs was 256.5 ± 9.2 × 5.5 ± 0.5. The microfilariae of *A. dracuculoides* are slightly shorter than *A. reconditum*, as reported in dogs in this study (Table 1) and in [13] as well as in foxes [14,15].

In recent publications [16,17] or guidelines [18], broader size ranges are reported. This could partly be due to the influence of examination methods. Air-drying and methanol fixation as used for Giemsa staining will affect size in a different way than the formalin treatment of the Knott’s technique. In a recent study, the mean length of Giemsa-stained *D. immitis* and *D. repens* mf in thin blood films [19] was shown to be significantly shorter than after applying the Knott’s test.

Other species of canine filariae, such as *Cercopithifilaria* spp., present microfilariae in the dermis, thus they do not represent a real issue in the differential diagnosis of mf circulating in blood. Furthermore, the length of *Cercopithifilaria* spp. mf significantly differs from the length of *Dirofilaria* and *Acanthocheilonema* spp. mf [16].

The Knott’s technique has several advantages. It is easy to perform, rapid and inexpensive, it conserves mf morphology and size, and it enhances the sensitivity of mf detection in blood samples. Courtney and Zeng [20] showed that only in 80.9% of the samples in which *D. immitis* mf had been detected by the Knott’s test, the parasites could also be found in direct smears and that the sensitivity of the direct smear was especially low in samples with less than 10 mf per ml. This is of particular relevance for *A. dracunculoides* and *A. reconditum* mf, since the average number of mf/ml is usually low in these species [10,11,13].

Considering the recent spread of canine filarial infections, particularly of *D. repens*, from southern to more northern and eastern European areas [21,22], the increased importance of canine travel medicine due to tourism with pets and the import of dogs from eastern and southern endemic European countries to central Europe [23], the diagnosis of filarial infections in dogs is becoming more and more important.

## Conclusion

The morphometric analysis proved to be a very useful, quick and inexpensive diagnostic tool and it represents the first step in the diagnosis of filarial infections. However, the discrimination between different species can be challenging in cases of mixed infections [17,24] or in cases with low parasitaemia. In these cases, as well as in cases where discrimination between *Acanthocheilonema* spp. is required, molecular methods (e. g. PCR [7,25]) or histochemical staining [5,6] are required.

## Competing interests

The authors declare that they have no competing interests.

## Authors’ contributions

JM participated in the design of the study and coordinated the sample collection; this paper represents his doctoral thesis. SL participated with the laboratory analyses. LG contributed with the sample collection and preparation of the manuscript. FG participated with the laboratory analyses and preparation of the manuscript. MM contributed with the sample collection and drafted the manuscript. TJN enabled the sample collection and participated with the design of the study. PD conceived the study and implemented the draft of the manuscript. All authors read and approved the final version of the manuscript.
